# Risk and outcomes of diabetes in patients with epilepsy

**DOI:** 10.1038/s41598-021-98340-x

**Published:** 2021-09-23

**Authors:** Chun-Cheng Li, Chuen-Chau Chang, Yih-Giun Cherng, Chao-Shun Lin, Chun-Chieh Yeh, Yi-Cheng Chang, Chaur-Jong Hu, Chun-Chuan Shih, Ta-Liang Chen, Chien-Chang Liao

**Affiliations:** 1 Department of Anesthesiology, Shuang Ho Hospital, Taipei Medical University, New Taipei City, Taiwan; 2 Department of Anesthesiology, School of Medicine, College of Medicine, Taipei Medical University, Taipei, Taiwan; 3 Department of Anesthesiology, Taipei Medical University Hospital, Taipei Medical University Hospital, 252 Wuxing St., Taipei, 110 Taiwan; 4 Anesthesiology and Health Policy Research Center, Taipei Medical University Hospital, Taipei, Taiwan; 5 Department of Surgery, China Medical University Hospital, Taichung, Taiwan; 6 Department of Surgery, University of Illinois, Chicago, USA; 7 Division of Endocrinology, Department of Internal Medicine, National Taiwan University Hospital, Taipei, Taiwan; 8 Graduate Institute of Medical Genomics, National Taiwan University, Taipei, Taiwan; 9 Institute of Biomedical Sciences, Academia Sinica, Taipei, Taiwan; 10 Department of Neurology, Shuang Ho Hospital, Taipei Medical University, New Taipei City, Taiwan; 11 School of Chinese Medicine for Post-Baccalaureate, I-Shou University, Kaohsiung City, Taiwan; 12 Department of Anesthesiology, Wan Fang Hospital, Taipei Medical University, Taipei, Taiwan; 13 Research Center of Big Data and Meta-Analysis, Wan Fang Hospital, Taipei Medical University, Taipei, Taiwan; 14 School of Chinese Medicine, College of Chinese Medicine, China Medical University, Taichung, Taiwan

**Keywords:** Diseases, Endocrinology, Health care, Risk factors

## Abstract

The risk and outcomes of diabetes in patients with epilepsy remains unclear. We evaluated these risks using an epilepsy cohort analysis and a diabetes admission analysis. In the epilepsy cohort analysis, we identified 2854 patients with newly diagnosed epilepsy in 2000–2008 from the research data of National Health Insurance in Taiwan. Using Propensity-score matching by sociodemographic factors and medical conditions, we selected 22,832 people without epilepsy as a non-exposed cohort for comparison. Follow-up events of diabetes from January 1, 2000 until December 31, 2013 were ascertained from medical claims. The adjusted hazard ratios (HRs) and 95% confidence intervals (CIs) of diabetes associated with epilepsy were calculated using multiple Cox proportional hazard models. In the diabetes admission analysis, we identified 92,438 hospitalized diabetes patients, 930 of whom had a history of epilepsy. Adjusted odds ratios (ORs) and 95% CIs of adverse events after diabetes associated with previous epilepsy were calculated using multiple logistic regressions. The adjusted HR of diabetes in the cohort with epilepsy was 1.31 (95% CI 1.14–1.50) compared to the non-epilepsy cohort. Previous epilepsy was associated with post-diabetes adverse events, such as pneumonia (OR 1.68, 95% CI 1.37–2.07), urinary tract infection (OR 1.83, 95% CI 1.55–2.16), and septicemia (OR 1.34, 95% CI 1.09–1.65). In conclusion, epilepsy was associated with higher risk of diabetes and adverse post-diabetes outcomes. Diabetes prevention and attention to post-diabetes adverse events are needed for this susceptible population.

## Introduction

Epilepsy is a disorder of the brain characterized by an enduring predisposition to seizures^1^. In the United States, approximately 2.5 million people have epilepsy, and it contributes to $12.5 billion in direct and indirect costs each year^2^. Although the cause of epilepsy is not completely understood, some risk factors have been identified, such as genetic conditions, abnormalities in brain development, stroke, head injuries, infections, tumors, or brain damage during or after birth^3^.

Diabetes is the most common metabolic and endocrine disease, affecting 6.9% of people in the United States^4^. It has been estimated that at least 20% of American adults over the age of 18 will have diabetes by the year 2050^5^. Microvascular and macrovascular complications are the major causes of death and disability in people with diabetes^6,7^. Diabetes is also one of the determinants for perioperative complications and mortality in both cardiac and non-cardiac surgeries^8,9^.

Previous studies have shown that patients with diabetes have a higher predisposition to develop epilepsy^10,11^. In contrast, the risk of diabetes in patients with epilepsy is not completely understood due to limitations of previous investigations, such as inappropriate study design, small sample size, and inadequate adjustment for confounding factors^12–16^. In addition, the impact of epilepsy on the outcomes after diabetes has not been validated. Using the reimbursement claims of the Taiwan National Health Research Database, we conducted this study included two analyses to investigate the risk and outcomes of diabetes in patients with epilepsy.

## Methods

### Source of data

In this study, we used the research data of the representative sample included one million persons in the Taiwan’s National Health Insurance. Details of this database were described in the previous studies^9,17–19^. There is no direct human participation in this study. Our study was approved by the Joint Institutional Review Boards of Taipei Medical University (TMU-JIRB-201801059; TMU-JIRB-201701050; TMU-JIRB-201912046). The Joint Institutional Review Boards of Taipei Medical University waived the need for informed consent of this study. All methods were carried out in accordance with relevant guidelines and regulations.

### Study design

There were two analyses in the present investigation. In Analysis I (the retrospective cohort analysis), we excluded data from individuals with previous medical histories of diabetes and/or epilepsy from 1996 to 1999 in the cohort included 1,000,000 persons. The cohort with incident epilepsy included 2854 adults aged ≥ 20 years who were identified in the 2000–2008. These patients had at least one visit of outpatient or inpatient care for epilepsy. We used propensity-score matching procedure by sociodemographic factors and medical conditions at the same study time interval to select 22,832 adults as the cohort without epilepsy and all of them had no medical history of epilepsy. Therefore, members of both the cohorts with and without epilepsy had no history of diabetes at the beginning of follow-up period. We started the follow-up period in 2000 and it continued until the end of 2013. Incident cases of diabetes were identified during the follow-up period. The aim of this analysis was to evaluate the risk of diabetes in patients with epilepsy.

In Analysis II, we identified 92,438 patients with admission of types 2 diabetes admission in 2004–2013, 930 of them had epilepsy previously. We compared the short-term outcomes between diabetes patients with and without epilepsy including complications, the consumption of medical resources, and the case fatality within 30 days during or after hospitalization.

### Definitions and measurements

People with low income were identified according to the definition of the Ministry of Health and Welfare, Taiwan. Physicians’ diagnostic codes from the *International Classification of Diseases, Ninth Revision, Clinical Modification* (ICD-9-CM) were used to define diseases and morbidities in this study, such as diabetes (250) and epilepsy (345). Coexisting medical conditions included mental disorders (290–319), hypertension (401–405), chronic obstructive pulmonary disease (491, 492, and 496), head injury (800–804 and 850–854), stroke (430–438), ischemic heart disease (410–414), asthma, hyperlipidemia (272.0, 272.1, and 272.2), liver cirrhosis (571.2, 571.5, and 571.6), heart failure (428), and atrial fibrillation (427.3). Post-diabetes complications included septicemia (038 and 998.5), pneumonia (480–486) and urinary tract infection (599.0). Renal dialysis included hemodialysis and peritoneal dialysis were also recorded by the administrative code (D8 and D9).

### Statistical analysis

In Analysis I, we used a propensity-score matching procedure (exposure vs nonexposure ratio = 1:8) to select patients with and without epilepsy. A non-parsimonious multivariable logistic regression model was used to estimate a propensity score for patients with and without epilepsy. Clinical significance guided the initial choice of covariates in this model to include age, sex, low income, mental disorders, hypertension, chronic obstructive pulmonary disease, traumatic brain injury, stroke, ischemic heart disease, asthma, hyperlipidemia, liver cirrhosis and heart failure. We matched people with epilepsy to people without epilepsy using a greedy matching algorithm (without replacement) with a caliper width of 0.2 SD of the log odds of the estimated propensity score. The chi-square tests were used to examine the distributions of categorical data, including such as (age, sex, low income, and history of diseases) between people with and without epilepsy. Using the multiple Cox proportional hazards models, the hazard ratios (HRs) with 95% confidence intervals (CIs) for diabetes risk in the cohort with epilepsy were calculated with the adjustment of all covariates. The stratified analyses were used to evaluate the risk of diabetes in patients with epilepsy within subgroups.

In Analysis II, differences in the balances of age, sex, low income, history of diseases, and medications between diabetes patients with and without epilepsy were examined using chi-square tests. We performed multiple logistic regressions to estimate odds ratios (ORs) and 95% CIs of complications and mortality after diabetes in patients with epilepsy by adjusting for sociodemographic factors and history of diseases. All significance tests were two-sided using *p* < 0.05 as the level of significance. All data analyses were performed with SAS, version 9.1 (SAS Institute Inc., Cary, NC, USA) statistical software.

## Results

The distributions of age, sex, low income, mental disorders, hypertension, chronic obstructive pulmonary disease, traumatic brain injury, stroke, ischemic heart disease, asthma, hyperlipidemia, liver cirrhosis and heart failure were balanced between cohorts with and without epilepsy because propensity-score matching was used in the Analysis I (Table 1).

**Table 1:** Characteristics of study subjects with and without epilepsy.

	Epilepsy				
	No (N = 22,832)		Yes (N = 2854)		
	n	(%)	n	(%)	
Age, years					
20–29	5928	(26.0)	5928	(26.0)	> 0.9999
30–39	3968	(17.4)	3968	(17.4)	
40–49	4360	(19.1)	4360	(19.1)	
50–59	2864	(12.5)	2864	(12.5)	
60–69	2152	(9.4)	2152	(9.4)	
70–79	2528	(11.1)	2528	(11.1)	
≥ 80	1032	(4.5)	1032	(4.5)	
Sex					> 0.9999
Female	10,320	(45.2)	10,320	(45.2)	
Male	12,512	(54.8)	12,512	(54.8)	
Low income	520	(2.3)	520	(2.3)	> 0.9999
Coexisting medical conditions*					
Mental disorders	11,480	(50.3)	11,480	(50.3)	> 0.9999
Hypertension	6256	(27.4)	6256	(27.4)	> 0.9999
COPD	4656	(20.4)	4656	(20.4)	> 0.9999
Traumatic brain injury	3496	(15.3)	3496	(15.3)	> 0.9999
Stroke	1760	(7.7)	1760	(7.7)	> 0.9999
Ischemic heart disease	3240	(14.2)	3240	(14.2)	> 0.9999
Asthma	2408	(10.6)	2408	(10.6)	> 0.9999
Hyperlipidemia	1440	(6.3)	1440	(6.3)	> 0.9999
Liver cirrhosis	488	(2.1)	488	(2.1)	> 0.9999
Heart failure	248	(1.1)	248	(1.1)	> 0.9999

*COPD* chronic obstructive pulmonary disease.

*After propensity-score matching procedure, both groups have no atrial fibrillation or renal dialysis.

The cohort with epilepsy had a higher incidence of diabetes than the non-epilepsy cohort (10.3 vs. 8.19 per 1000 person-years, *P* < 0.0001) and the corresponding HR of diabetes associated with epilepsy was 1.31 (95% CI 1.14–1.50) during the follow-up period (Table 2). In the subgroup analysis, the association between epilepsy and risk of diabetes was significant in women (HR 1.23, 95% CI 1.01–1.51), men (HR 1.38, 95% CI 1.15–1.65), and those aged 30–39 years (HR 1.66, 95% CI 1.09–2.52), 50–59 years (HR 1.45, 95% CI 1.10–1.91), and 70–79 years (HR 1.45, 95% CI 1.04–2.00).

**Table 2:** Risk of diabetes in association with epilepsy stratified by age and sex.

		n	Events	Person-years	Incidence^†^	HR	(95% CI)*
No epilepsy		22,832	1715	209,303	8.19	1.00	(Reference)
Epilepsy		2854	269	26,223	10.3	1.31	(1.14–1.50)
Female	No epilepsy	10,320	826	94,457	8.74	1.00	(Reference)
	Epilepsy	1290	126	11,916	10.6	1.23	(1.01–1.51)
Male	No epilepsy	12,512	889	114,847	7.74	1.00	(Reference)
	Epilepsy	1564	143	14,307	10.0	1.38	(1.15–1.65)
Age, 20–29 years	No epilepsy	5928	137	55,775	2.46	1.00	(Reference)
	Epilepsy	741	23	7150	3.22	1.07	(0.64–1.79)
Age, 30–39 years	No epilepsy	3968	146	37,130	3.93	1.00	(Reference)
	Epilepsy	496	29	4700	6.17	1.66	(1.09–2.52)
Age, 40–49 years	No epilepsy	4360	409	39,760	10.3	1.00	(Reference)
	Epilepsy	545	64	5023	12.7	1.26	(0.96–1.67)
Age, 50–59 years	No epilepsy	2864	399	25,460	15.7	1.00	(Reference)
	Epilepsy	358	64	2876	22.3	1.45	(1.10–1.91)
Age, 60–69 years	No epilepsy	2152	319	18,966	16.8	1.00	(Reference)
	Epilepsy	269	40	2471	16.2	1.00	(0.71–1.42)
Age, 70–79 years	No epilepsy	2528	259	22,651	11.4	1.00	(Reference)
	Epilepsy	316	42	2848	14.7	1.45	(1.04–2.00)
Age, ≥ 80 years	No epilepsy	1032	46	9561	4.81	1.00	(Reference)
	Epilepsy	129	7	1156	6.06	1.39	(0.63–3.09)

*CI* confidence interval, *HR* hazard ratio.

*Adjusted for all covariates listed in Table 1.

^†^Per 1000 person-years.

Among the 92,438 patients with diabetes admission (Table 3), patients with epilepsy had higher proportions of males (*P* < 0.0001), older people (*P* < 0.0001), low income (*P* < 0.0001), stay in medical center (*P* < 0.0001), mental disorders (*P* < 0.0001), stroke (*P* < 0.0001), chronic obstructive pulmonary disease (*P* < 0.0001), ischemic heart disease (*P* = 0.001), traumatic brain injury (*P* < 0.0001), liver cirrhosis (*P* < 0.0001), asthma (*P* = 0.0476), congestive heart failure (*P* = 0.0314), and atrial fibrillation (*P* = 0.0156) compared with non-epilepsy patients.

**Table 3:** Characteristics of hospitalized diabetes patients with and without previous epilepsy.

	Epilepsy*				*P*
	No (N = 91,508)		Yes (N = 930)		
	n	(%)	n	(%)	
Age, years					
20–29	1011	(1.1)	17	(1.8)	< 0.0001
30–39	3108	(3.4)	64	(6.9)	
40–49	8745	(9.6)	131	(14.1)	
50–59	18,712	(20.5)	181	(19.5)	
60–69	22,201	(24.3)	173	(18.6)	
70–79	25,109	(27.4)	225	(24.2)	
≥ 80	12,622	(13.8)	139	(15.0)	
Sex					< 0.0001
Female	43,532	(47.6)	349	(37.5)	
Male	47,976	(52.4)	581	(62.5)	
Low income	4990	(5.4)	107	(11.5)	< 0.0001
Stay in Medical center	29,963	(32.7)	221	(23.8)	< 0.0001
Coexisting medical conditions					
Hypertension	34,462	(37.7)	322	(34.6)	0.0572
Mental disorders	18,326	(20.0)	307	(33.0)	< 0.0001
Stroke	9090	(9.9)	195	(21.0)	< 0.0001
COPD	11,907	(13.0)	186	(20.0)	< 0.0001
Ischemic heart disease	17,381	(19.0)	137	(14.7)	0.0010
Traumatic brain injury	4541	(5.0)	130	(14.0)	< 0.0001
Renal dialysis	10,452	(11.4)	104	(11.2)	0.8195
Liver cirrhosis	4319	(4.7)	75	(8.1)	< 0.0001
Asthma	5207	(5.7)	67	(7.2)	0.0476
Heart failure	7262	(7.9)	56	(6.0)	0.0314
Hyperlipidemia	5187	(5.7)	44	(4.7)	0.2184
Atrial fibrillation	796	(0.9)	15	(1.6)	0.0156

*COPD* chronic obstructive pulmonary disease.

*New-diagnosed diabetes after epilepsy.

Table 4 shows that patients with epilepsy had a higher risk of urinary tract infection (OR 1.51, 95% CI 1.37–1.67), pneumonia (OR 1.68, 95% CI 1.37–2.07), and septicemia (OR 1.34, 95% CI 1.09–1.65) after diabetes admission compared with those without epilepsy in the Analysis I.

**Table 4:** Adverse outcomes after diabetes admission in patients with epilepsy*.

	No epilepsy, %	Epilepsy, %	OR	(95% CI)^†^
30-day in-hospital mortality	1.1	1.8	1.59	(0.98–2.60)
Urinary tract infection	14.2	22.2	1.83	(1.55–2.16)
Pneumonia	6.6	11.7	1.68	(1.37–2.07)
Septicemia	8.5	11.4	1.34	(1.09–1.65)
Medical expenditure, USD	3035 ± 4589	3237 ± 4923	*P* = 0.2150	
Length of hospital stay, days	17.4 ± 26.6	18.0 ± 23.9	*P* = 0.4743	

*CI* confidence interval, *OR* odds ratio, *USD* United States dollars.

*New-diagnosed diabetes after epilepsy.

^†^Adjusted for all covariates listed in Table 3.

The supplemental file (Table S1) demonstrates that the risk of diabetes was more significant in patients with epilepsy who had alcohol-related illness (HR 1.64, 95% CI 1.12–2.40). The epilepsy-related clinical indicators also had impacts on post-diabetes adverse events, such as generalized seizure (OR 1.42, 95% CI 1.02–1.98), low income (OR 1.80, 95% CI 1.18–2.74), emergency care (OR 1.81, 95% CI 1.41–2.34), and traumatic brain injury (OR 1.59, 95% CI 1.09–2.33).

## Discussion

Using the data from the Taiwan’s National Health Insurance, our Analysis I showed patients with epilepsy had an increased risk of diabetes during the follow-up period. In the Analysis II, we found the increased risk of pneumonia, urinary tract infection, and septicemia in patients with diabetes who had a history of epilepsy. Our investigation is the first population-based study showing the associated risk and outcomes of diabetes for patients with epilepsy.

Age, gender, and low income are known factors associated with diabetes^20–22^. Several medical conditions, such as hypertension, mental disorders, ischemic heart disease, chronic obstructive pulmonary disease, asthma, renal dialysis, and stroke also commonly coexist with diabetes^9^. The abovementioned diseases have also been identified as common comorbidities of epilepsy in previous studies^12–16^. These factors were potential confounders when analyzing the association between epilepsy and risk of diabetes. To avoid confounding bias when investigating this relationship, we adjusted these sociodemographic factors and coexisting medical conditions in the multivariate regression models.

There are some possible reasons that may explain why patients with epilepsy had increased risk and adverse outcomes of diabetes. First, it has been reported that patients with epilepsy have increased cortisol levels^23,24^. Generalized neuronal discharge of a seizure stimulates the hypothalamus either directly, through specific neurotransmitter changes, or through the release of other substances^24^. The activation of the hypothalamus–pituitary–adrenal axis causes elevated cortisol. Hypercortisolism is responsible for the occurrence of dyslipidemia, insulin intolerance, and diabetes^25^. Subclinical hypercortisolism may also contribute to the risk of diabetes^26^. Second, people with epilepsy exercise less and tend to be more obese^27^. Social stigma and concerns for injury discourage patients with epilepsy from exercise and activity. Concomitant somatic and psychological diseases also limit their participation in physical activity. Anti- epilepsy drugs can also stimulate appetite and cause lethargy, thereby contributing to weight gain^28^, which is a common risk factor for diabetes. Third, epilepsy is highly associated with psychiatric disease^13,14,16^. Depression and antipsychotic medications are known risk factors for diabetes^29,30^. Fourth, patients with epilepsy are more likely to have low socioeconomic status, which may limit their knowledge, attitude, and practice for caring for impaired glucose homeostasis^31^.

Our results showed epilepsy increases the risk of diabetes, which aligns with the findings of previous studies^12–16^. However, little is known about the effect of gender on the association between epilepsy and diabetes. In a further analysis of this study, we found that the impact of epilepsy on risk of diabetes is significant in both male and female populations. Moreover, alcohol-related illness, severe mental disorder, low income, and emergency care for epilepsy further increase the risk of diabetes. This finding is reasonable because individuals with those factors usually have poor epilepsy control^32^.

Our analysis also showed that patients with epilepsy have a higher risk of diabetes, except those who are older than 60 years of age. This phenomenon may be because the incidence of diabetes in the non- epilepsy group is lower among young people, therefore making the effect of epilepsy more prominent. Another possible explanation is that epilepsy severity decreases with age^33^, making the impact of epilepsy on other diseases less evident in the elderly group.

In the Analysis II investigating in-hospital adverse outcomes, we found that the numbers of post-diabetes infections in patients with epilepsy are significantly higher than those without epilepsy. Although these findings have not been reported previously, one study of adverse postoperative outcomes in patients with epilepsy showed similar results^17^. Epilepsy and related comorbidities are also responsible for increased risk of community-acquired pneumonia^34^. Epilepsy itself is known to increase the risk of aspiration pneumonia due to increased oral secretions, impaired swallowing mechanism, and difficulty in attaining adequate patient positioning^35^. Use of anti-epilepsy drugs also increases the risk of infection. Although the mechanism is unclear, anti-epilepsy drugs decrease the production of some proinflammatory cytokines, which might facilitate and worsen infection^36^. We considered that the possibility that the difference of adverse events after diabetes admission between the epilepsy group and the non-epilepsy group could simply reflect the difference of events between the epilepsy group and the non-epilepsy group not related to diabetes. This is because adverse events, such as pneumonia and urinary tract infection, are not specific events to diabetes.

We also found that patients with epilepsy who had low income, alcohol-related disease, severe mental disorder, emergency care for epilepsy, and traumatic brain injury were at an increased risk of diabetes. This is reasonable because those patients typically have poor self-care and epilepsy control.

To the best of our knowledge, the present study is the first investigations reporting that epilepsy increases the risk of diabetes and poor post-diabetes outcomes. Previously, it was known that patients with epilepsy face a mortality rate 1.4–9.7 times greater than that of the general population^37,38^. Causes of mortality include the underlying neurologic cause of epilepsy (stroke and cancer), sudden unexpected death in epilepsy, status epilepticus, consequence of seizure attack (accident, drowning, and aspiration pneumonia), suicide, and iatrogenic death^37,38^. The impact of diabetes on patients with epilepsy could be more pronounced than that of other diseases because poor glycemic control and hyperglycemia are themselves associated with the severity of seizures in human and animal studies^39,40^. This finding implies that epilepsy and diabetes have reciprocal effects and that poor control of both diseases can result in a vicious cycle.

This study has some limitations. First, the National Health Research Database lacks information on laboratory data, clinical examinations, socioeconomic factors, and lifestyle and thus those factors were not considered as covariates in this study. Second, the duration and severity of epilepsy are not available from the database. Thus, we could not analyze the severity-related effects of epilepsy on diabetes. Third, people with minor or well-controlled epilepsy may not consult a doctor. However, if epilepsy is associated with diabetes, this misclassification would lead to underestimation of the association between epilepsy and diabetes risk. In addition, although we adjusted for several major potential confounders, residual confounding effects are always possible. Finally, the use of retrospective data in this study could not confirm the causal inference for the association between epilepsy and diabetes risk. This study is based on the database in Taiwan, which means the generalizability to other races or countries is uncertain.

In conclusion, we investigated patients with epilepsy had an increased risk of diabetes and adverse post-diabetes outcomes. This study provided a comprehensive assessment of diabetes risk and short-term outcomes in patients with epilepsy. Since epilepsy is associated with diabetes and uncontrolled diabetes aggravates epilepsy, diabetes prevention is important in patients with epilepsy. Strategies for prevention such as education, healthier lifestyles, better access to medical service, and control of comorbidities are needed.

## Supplementary Information

Supplementary Table S1.

## Abbreviations

ICD-9-CM: International classification of diseases, 9th revision, clinical modification
CI: Confidence interval
OR: Odds ratio
HR: Hazard ratio
