# Supplementary material for: Risk and outcomes of diabetes in patients with epilepsy
Source: Sci Rep. 2021 Sep 23;11:18888. doi: 10.1038/s41598-021-98340-x (PMC8460720; doi:10.1038/s41598-021-98340-x)
Supplement: Supplementary file 1 — Supplementary Table S1. [file 41598_2021_98340_MOESM1_ESM.doc]

| **Table S1.** The impact of characteristics of epilepsy on diabetes risk and outcomes | | | | | | |
| --- | --- | --- | --- | --- | --- | --- |
|  | Risk of diabetes | | | Post-diabetes adverse events‡ | | |
| Characteristics epilepsy | n | HR | (95% CI)* | n | OR | (95% CI)† |
| No epilepsy | 22832 | 1.00 | (reference) | 91508 | 1.00 | (reference) |
| Epileptic patients with |  |  |  |  |  |  |
| Low income | 65 | 1.87 | (0.89-3.94) | 107 | 1.80 | (1.18-2.74) |
| Alcohol-related illness | 250 | 1.64 | (1.12-2.40) | 115 | 0.99 | (0.60-1.65) |
| Severe mental disorders | 407 | 1.28 | (0.92-1.79) | 113 | 1.03 | (0.65-1.63) |
| Emergency care for epilepsy | 583 | 1.06 | (0.77-1.44) | 282 | 1.81 | (1.41-2.34) |
| Traumatic brain injury | 437 | 0.99 | (0.67-1.45) | 130 | 1.59 | (1.09-2.33) |
| Generalized seizure | 738 | 1.16 | (0.89-1.52) | 178 | 1.42 | (1.02-1.98) |
| Inpatient care for epilepsy | 230 | 1.11 | (0.69-1.80) | 77 | 1.59 | (0.98-2.60) |
| Intractable epilepsy | 283 | 0.89 | (0.55-1.44) | 77 | 1.62 | (0.99-2.63) |
| *Multivariate adjusted in the Cox proportional hazard models.  †Multivariate adjusted in the logistic regression models  ‡Adverse events included urinary tract infection, pneumonia and septicemia.  CI, confidence interval; HR, hazard ratio. | | | | | | |
